# Supplementary material for: Structural Insights into Bortezomib-Induced Activation of the Caseinolytic Chaperone-Protease System in Mycobacterium tuberculosis
Source: Nat Commun. 2025 Apr 11;16:3466. doi: 10.1038/s41467-025-58410-4 (PMC11992174; doi:10.1038/s41467-025-58410-4)
Supplement: Supplementary file 1 — Supplementary Information [file 41467_2025_58410_MOESM1_ESM.pdf]

Supplementary Information

Structural Insights into Bortezomib-Induced Activation of the Caseinolytic Chaperone-Protease System in *Mycobacterium tuberculosis*

Zhou et al

Supplementary Information includes:

Supplementary Figures 1-15

Supplementary Tables 1

Supplementary References

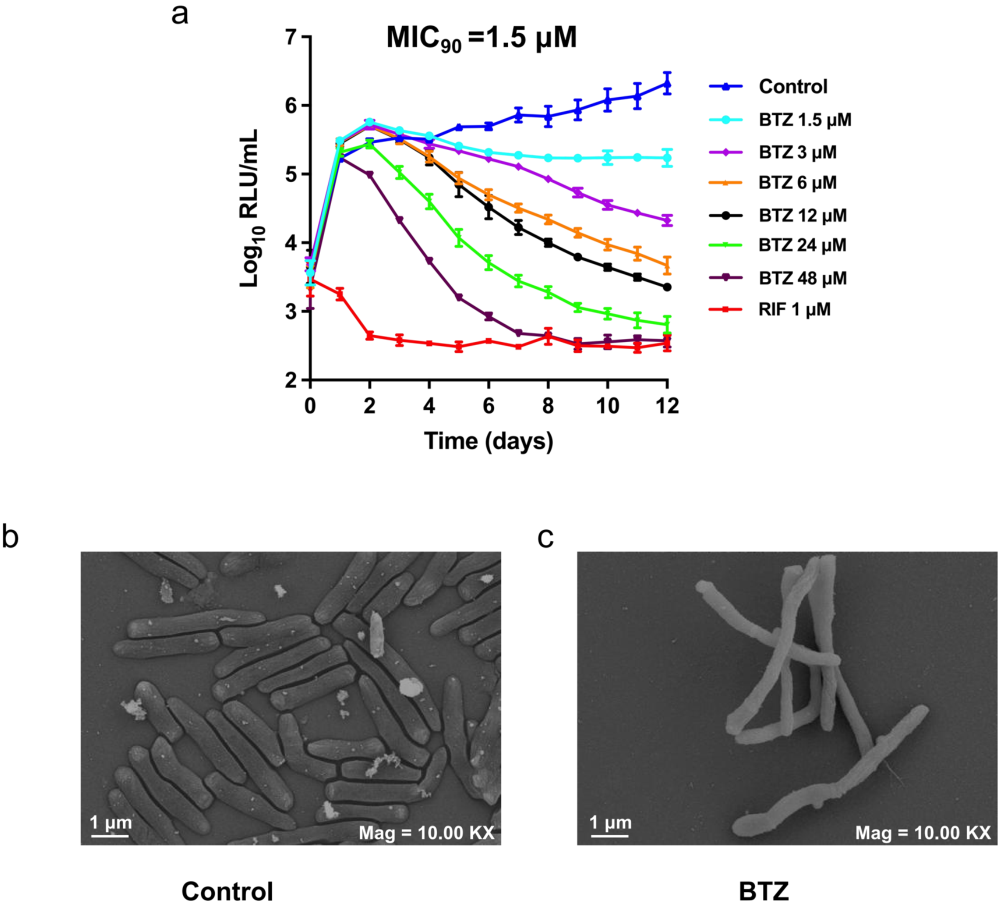

Supplementary Fig. 1 | Anti-mycobacterial activity of BTZ.

a, Anti-*Mtb* activity was measured using a previously established luminescence-based method[1] (see method section). The relative light units (RLU) of autoluminescent strains (*Mtb* H37Ra, UAlRa) were monitored after addition of indicated concentrations of BTZ for 12 consecutive days. DMSO as a negative control and rifampicin (RIF, 1 μM) as a positive control were tested. Data points are shown as mean ± SD from 3 independent experiments (n = 3). **b-c**, Scanning electron microscopy (SEM) showed that filamentous cells were formed after *Mycobacterium tuberculosis* cells were exposed to 6 μM BTZ for 12 hr (**c**) compared to non-exposed (control) cells (**b**). We repeated the experiment three times and obtained similar results. Source data are provided as a Source Data file.

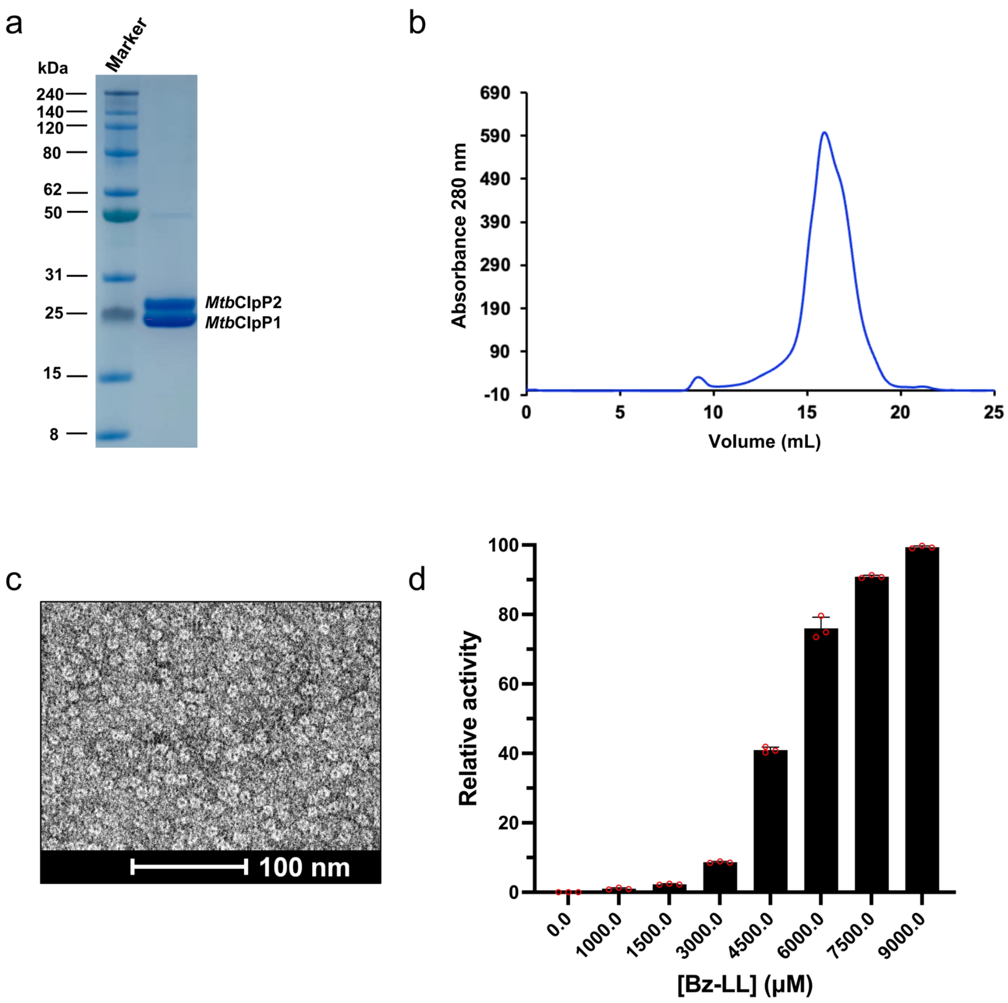

Supplementary Fig. 2 | Purification and proteolytic activity of the *Mtb*ClpP1P2 complex.

a, SDS-PAGE analysis of the purified *Mtb*ClpP1P2 complex. **b**, Size-exclusion chromatography profile of the purified *Mtb*ClpP1P2 complex. Both *Mtb*ClpP1 and *Mtb*ClpP2 are co-eluted at 15.6 mL (Superose^{TM} 6 increase 10/300 GL column) with an estimated molecular weight of ~300 kDa. **c**, Negative stain electron microscopy image of the purified *Mtb*ClpP1P2 complex. We repeated the experiment three times and obtained similar results. **d**, Peptidase activity of the *Mtb*ClpP1P2 tetradecamer complex (0.2 μM) against the small peptide substrate PMK-AMC (200 μM) in the presence of Bz-LL at the indicated concentrations. In each experiment, peptidase activities are normalized to the

highest activity. Histogram bars are shown as mean ± SD from 3 independent experiments (n = 3). Source data are provided as a Source Data file.

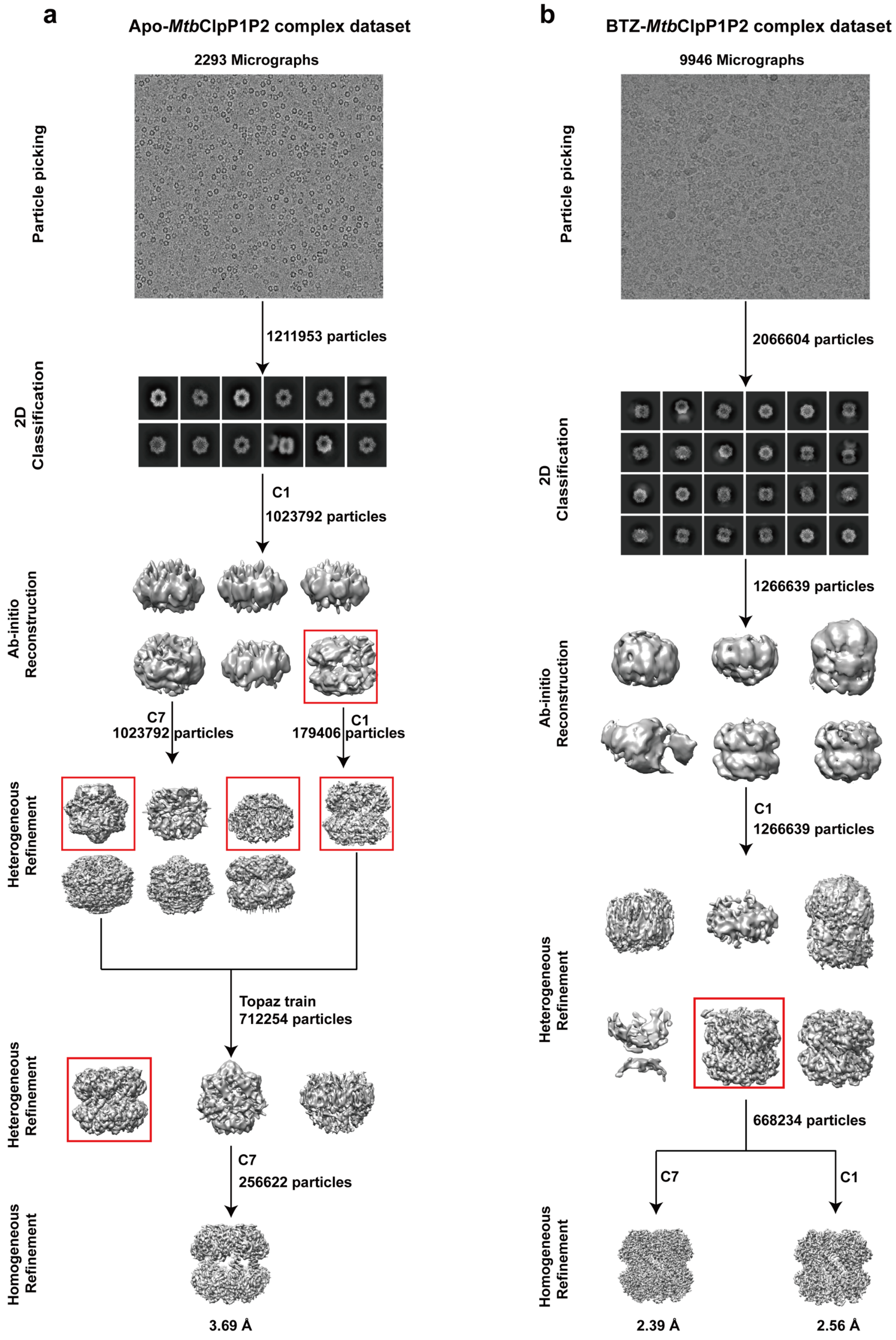

Supplementary Fig. 3 | Cryo-EM data processing pipelines for the *Mtb*ClpP1P2 complex datasets.

a, Data processing pipeline for the apo-*Mtb*ClpP1P2 complex dataset. **b**, Data processing pipeline for the BTZ-*Mtb*ClpP1P2 complex dataset. Red boxes indicate selected classes.

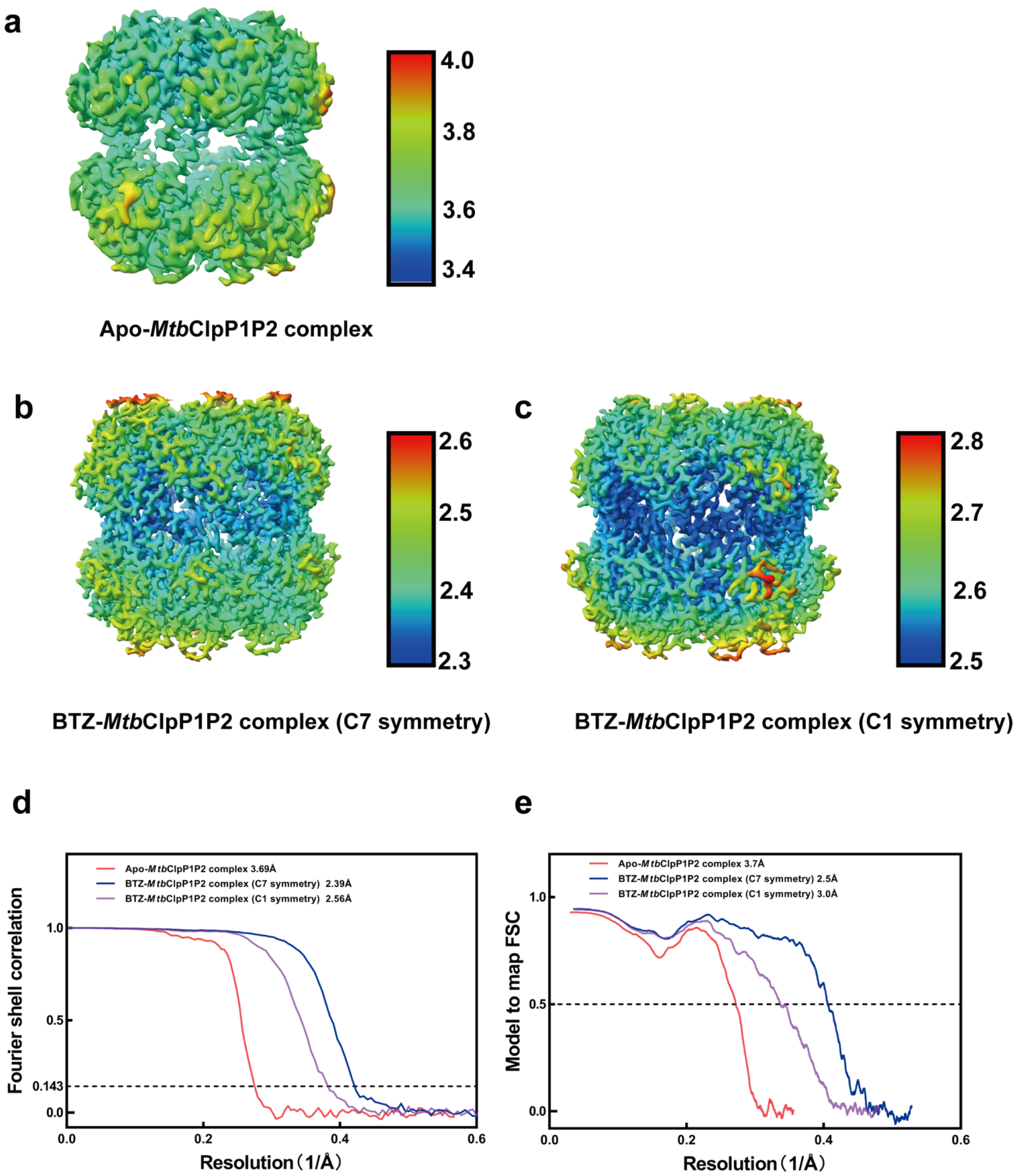

Supplementary Fig. 4 | Resolution assessment of the *Mtb*ClpP1P2 cryo-EM structures.

a-c, Local resolution assessments for the apo-*Mtb*ClpP1P2 **(a)** and BTZ-*Mtb*ClpP1P2 structures, imposing C7 **(b)** and C1 **(c)** symmetry. **d**, Global resolution assessment by Fourier shell correlation (FSC) at the 0.143 criterion. **e**, Model to map FSC at the 0.5 criterion.

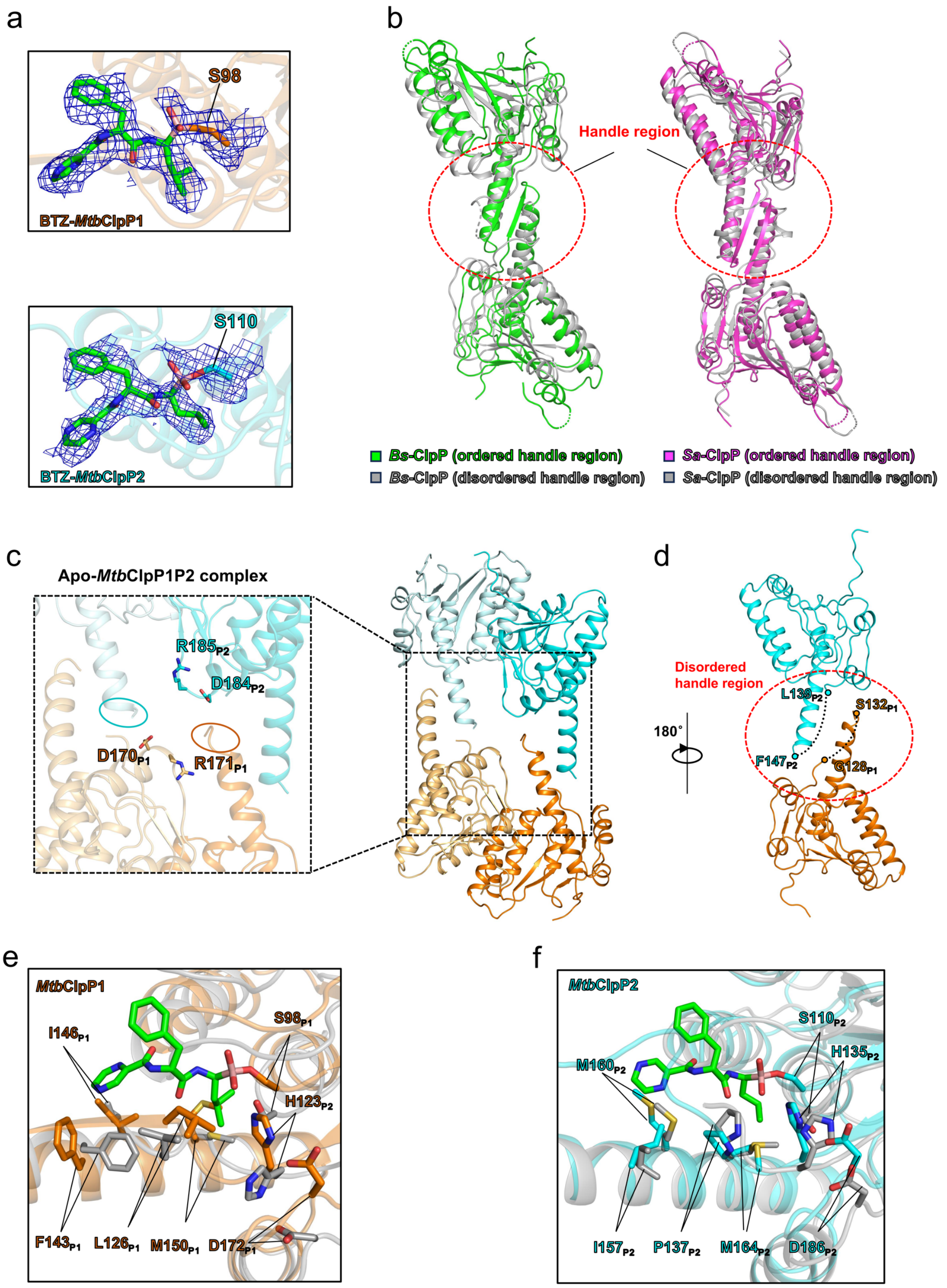

Supplementary Fig. 5 | Structural features of the BTZ-*Mtb*ClpP1P2 complex.

a, Blue meshes showing the densities of active site bound BTZ in the BTZ-*Mtb*ClpP1P2 complex structure. **b**, Overlaps of *Staphylococcus aureus* ClpP[2,3] (*Sa*ClpP, PDB code 3V5E and 5DL1) and *Bacillus subtilis* ClpP[4] (*Bs*ClpP, PDB

code 7FEQ and 7FER) structures showing the ordered and disordered handle regions. **c**, Cartoon representation of two apo *Mtb*ClpP1-ClpP2 dimers extracted from the tetradecameric apo-*Mtb*ClpP1P2 complex (right panel). The BTZ interacting region is shown in the black dotted box (left panel). **d**, Cartoon representation of one *Mtb*ClpP1-ClpP2 dimers extracted from the tetradecameric apo-*Mtb*ClpP1P2 complex in a different view from **c**. The disordered handle regions (res. ${}_{128}$GVTGS${}_{132}$ in ClpP1 and ${}_{139}$LSGVIQGQF${}_{147}$ in ClpP2) are highlighted within the red dotted ellipse. **e-f**, Cartoons showing *Mtb*ClpP1 (**e**) and *Mtb*ClpP2 (**f**) BTZ interacting residues in the BTZ-*Mtb*ClpP1P2 (colored) and apo-*Mtb*ClpP1P2 (grey) complexes.

77

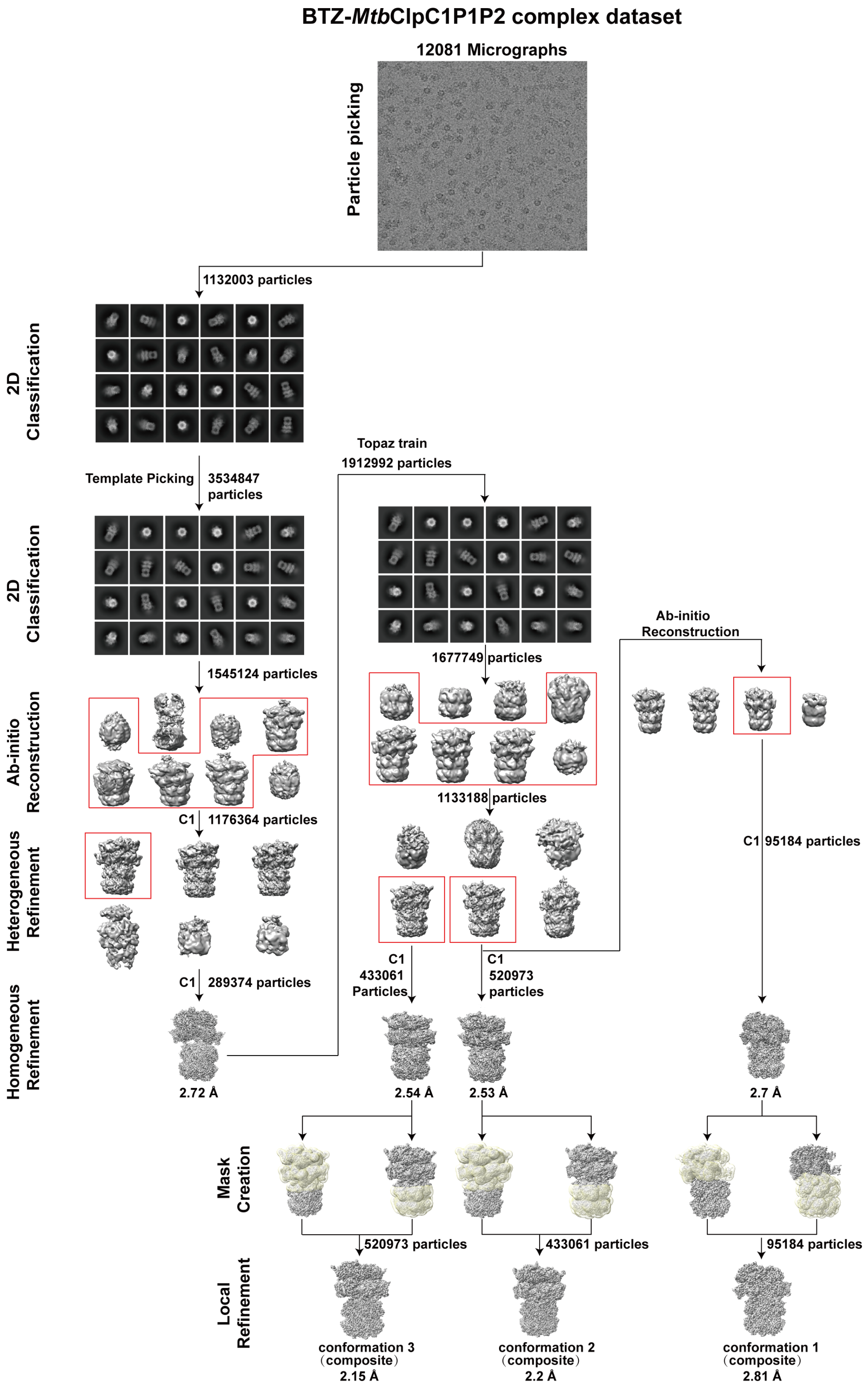

BTZ-MtbClpC1P1P2 complex dataset
12081 Micrographs
Particle picking
1132003 particles
2D Classification
Template Picking
3534847 particles
2D Classification
Topaz train
1912992 particles
1545124 particles
Ab-initio Reconstruction
1677749 particles
Ab-initio Reconstruction
C1 1176364 particles
1133188 particles
Heterogeneous Refinement
C1 95184 particles
C1 289374 particles
C1 433061 Particles
C1 520973 particles
Homogeneous Refinement
2.72 Å
2.54 Å
2.53 Å
2.7 Å
Mask Creation
520973 particles
433061 particles
95184 particles
Local Refinement
conformation 3 (composite) 2.15 Å
conformation 2 (composite) 2.2 Å
conformation 1 (composite) 2.81 Å

Supplementary Fig. 6 | Cryo-EM data processing pipeline for the BTZ-*Mtb*ClpC1P1P2 complex dataset.

Data processing pipeline for the BTZ-*Mtb*ClpC1P1P2 complex dataset. The masks for subtraction are shown as yellow transparent surfaces. Red boxes indicate selected classes.

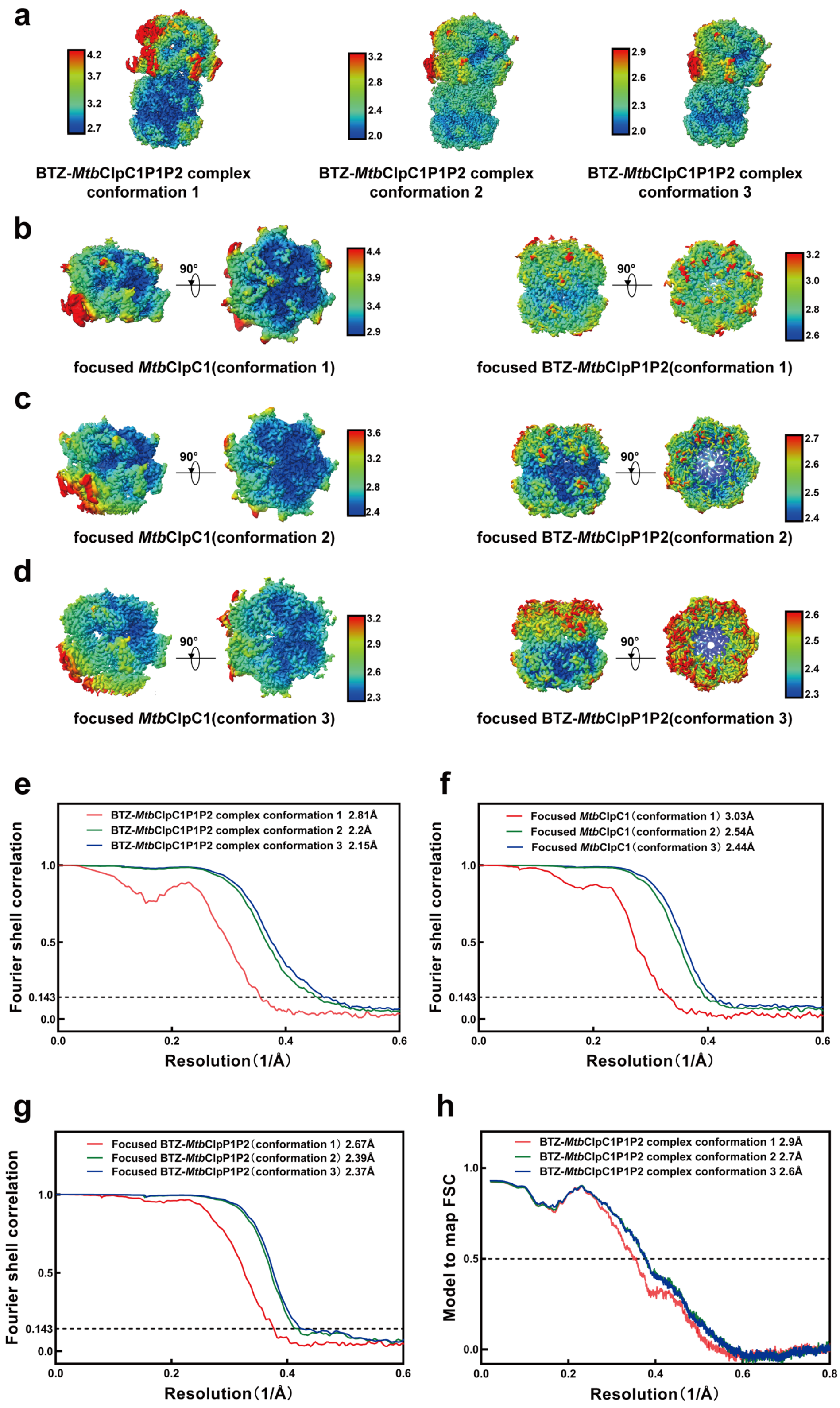

Supplementary Fig. 7 | Resolution assessment of the BTZ-*Mtb*ClpC1P1P2 complex cryo-EM structures.

a, Local resolution assessment for the BTZ-*Mtb*ClpC1P1P2 complex conformation 1 (left panel), conformation 2 (middle panel) and conformation 3 (right panel). **b**, Local resolution assessment for the BTZ-*Mtb*ClpC1P1P2 complex conformation 1 focused on *Mtb*ClpC1 (left panel) and BTZ-*Mtb*ClpP1P2 (right panel). **c**, Local resolution assessment for the BTZ-*Mtb*ClpC1P1P2 complex conformation 2 focused on *Mtb*ClpC1 (left panel) and BTZ-*Mtb*ClpP1P2 (right panel). **d**, Local resolution assessment for the BTZ-*Mtb*ClpC1P1P2 complex conformation 3 focused on *Mtb*ClpC1 (left panel) and BTZ-*Mtb*ClpP1P2 (right panel). **e-g**, Global resolution assessment by Fourier shell correlation (FSC) at the 0.143 criterion. **h**, Correlations of model vs map by FSC at the 0.5 criterion.

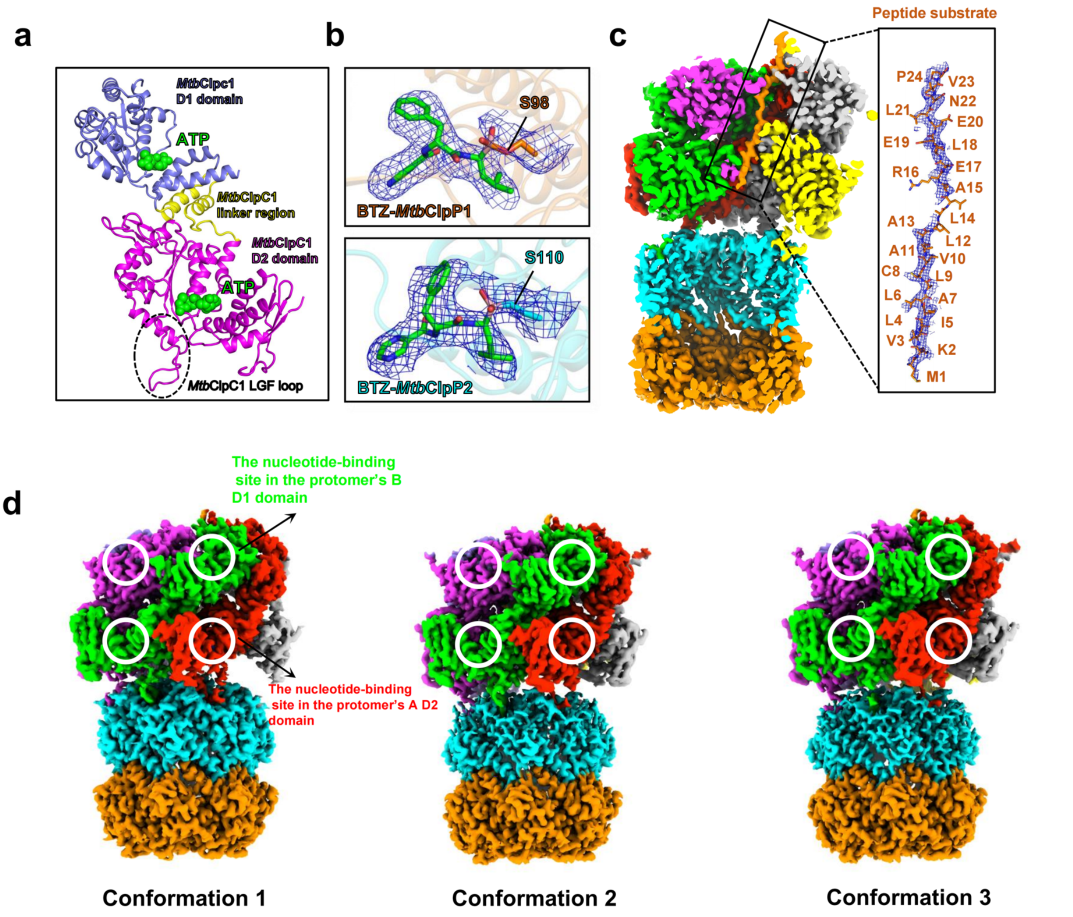

Supplementary Fig. 8 | Structural features of the *Mtb*ClpC1P1P2 complex in the three determined conformations.

a, Structure of an *Mtb*ClpC1 monomer. D1 and D2 domains are in different colors; the D1-D2 linker region is colored yellow; ATP molecules are shown as green spheres; the LGF loop is highlighted within the black dashed ellipse. **b**, Blue meshes showing the densities of the active site bound BTZ in the *Mtb*ClpC1P1P2 complex (conformation 3). **c**, Location of the bound substrate peptide in the *Mtb*ClpC1P1P2 complex (conformation 3); Inset showing the cryo-EM density of the bound substrate peptide and the modelled peptide sequence. **d**, The Cryo-EM maps showing the D1 and D2 domain swapping within the *Mtb*ClpC1 hexamers. The D1 domain of the preceding protomer interacts with the D2 domain of the following protomer. D1 and D2 nucleotide-binding pockets are indicated by white circles. As a result of the domain

swapping, the D1 nucleotide-binding pocket of each preceding protomer is positioned directly above the D2 nucleotide-binding pocket of the following protomer.

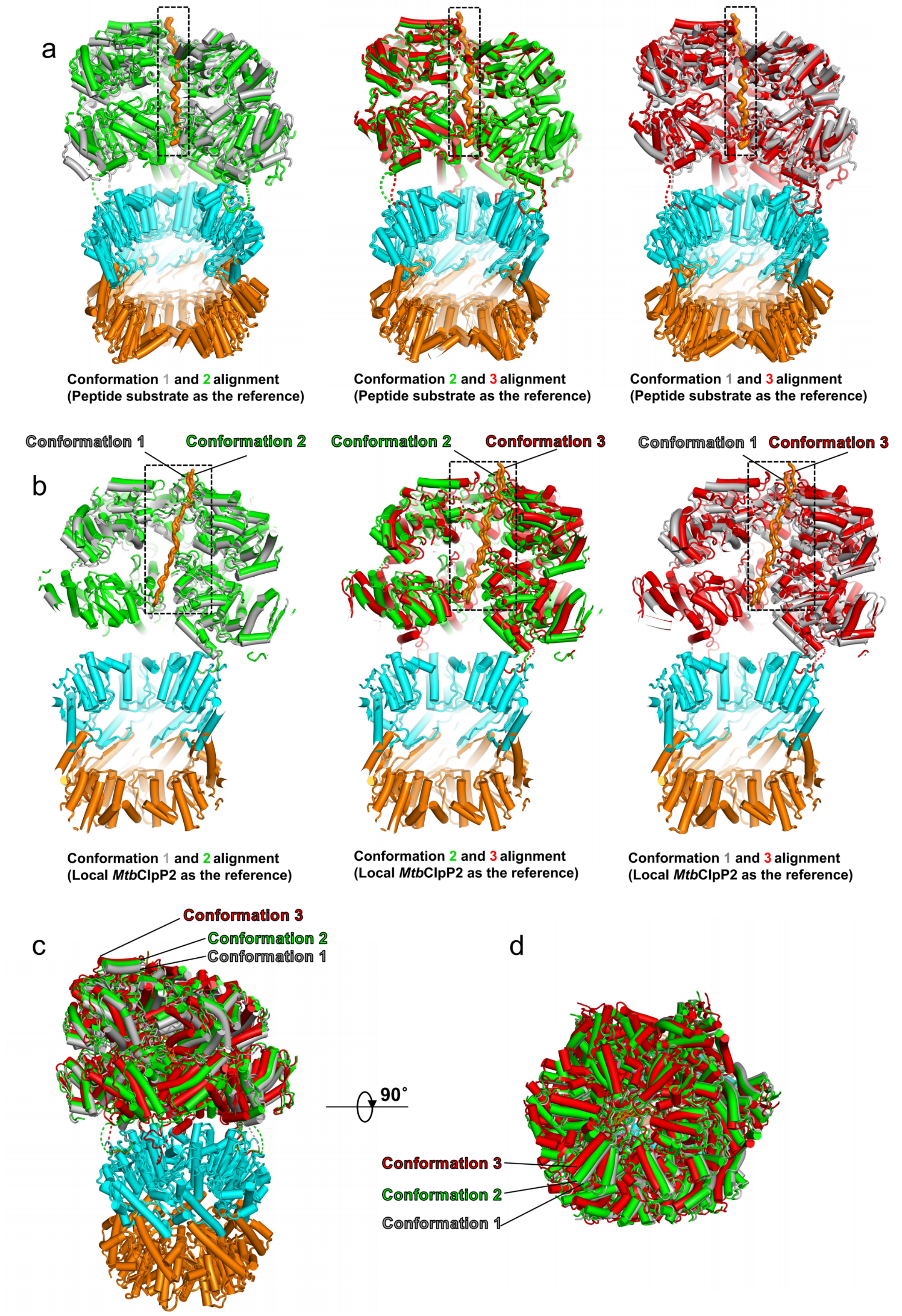

Supplementary Fig. 9 | Structural alignment of the *Mtb*ClpC1P1P2 complexes in three different conformations.

a, Sliced-through views of the pairwise alignments of the three *Mtb*ClpC1P1P2 structures in different conformations using the bound substrate peptide as the reference. **b**, Pairwise local alignments of the three *Mtb*ClpC1P1P2 structures based on the alignments in panel **a** and using *Mtb*ClpP2 as the reference. **c-d**, Side (**c**) and top (**d**) views showing the superposition of the three *Mtb*ClpC1P1P2 complexes based on the alignments in panel **b**.

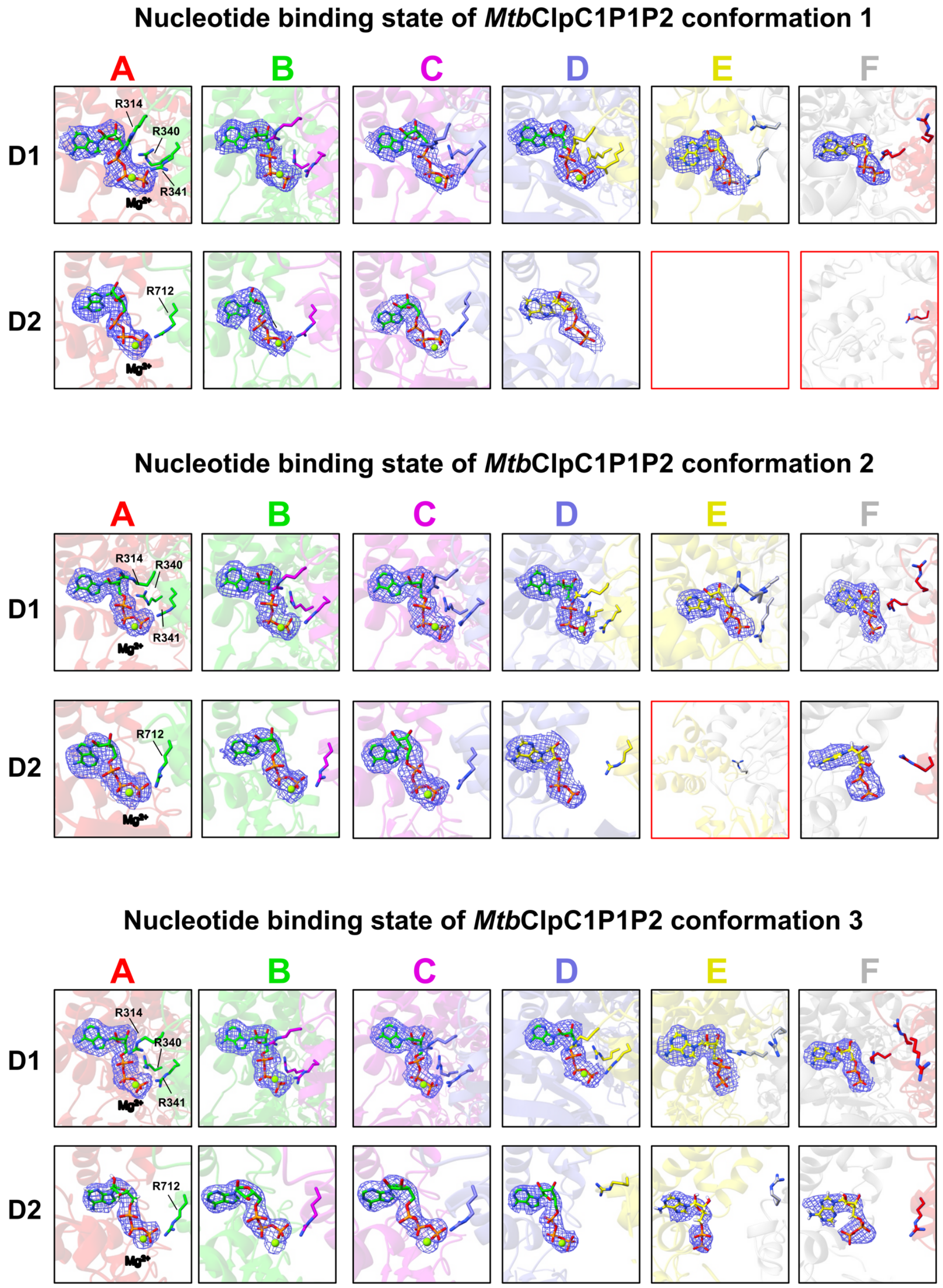

Supplementary Fig. 10 | Nucleotide densities in the three *Mtb*ClpC1P1P2 complexes.

Cryo-EM densities (blue meshes) of nucleotides bound at each nucleotide binding site in D1 and D2 rings in the three conformations. Nucleotide density is not observed in the D2 domains of protomers E and F in conformation 1, and D2 domain of protomer E in conformation 2. The green spheres represent

bound Mg^{2+} ions. The trans-activating Arg-finger residues (R340 and R341 in D1 ring; R712 in D2 ring) are shown in stick representations. D1 domain residue R314, a key nucleotide interacting residue, is also shown.

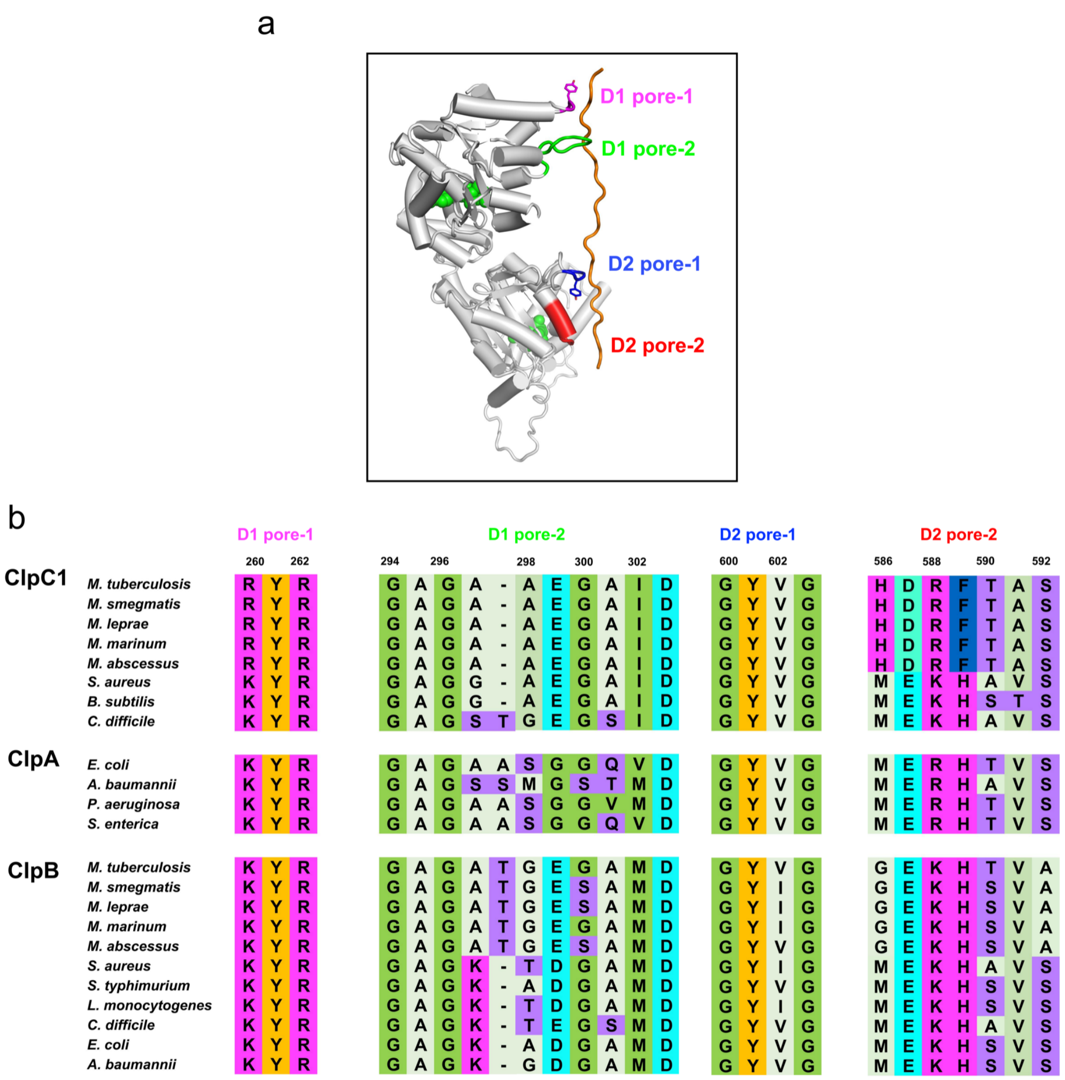

Supplementary Fig. 11 | The conserved pore motifs in *Mtb*ClpC1.

a, Locations of the D1 pore-1 loop (magenta), D1 pore-2 loop (green), D2 pore-1 loop (blue), and D2 pore-2 loop (red) in protomer A of the substrate peptide (orange) bound ClpC1 hexamer. **b**, Multiple sequence alignment of D1 pore-1 loop, D1 pore-2 loop, D2 pore-1 loop, and D2 pore-2 loop sequences across ClpABC family members. Amino acids that deviate from the consensus sequence are highlighted with contrasting colors.

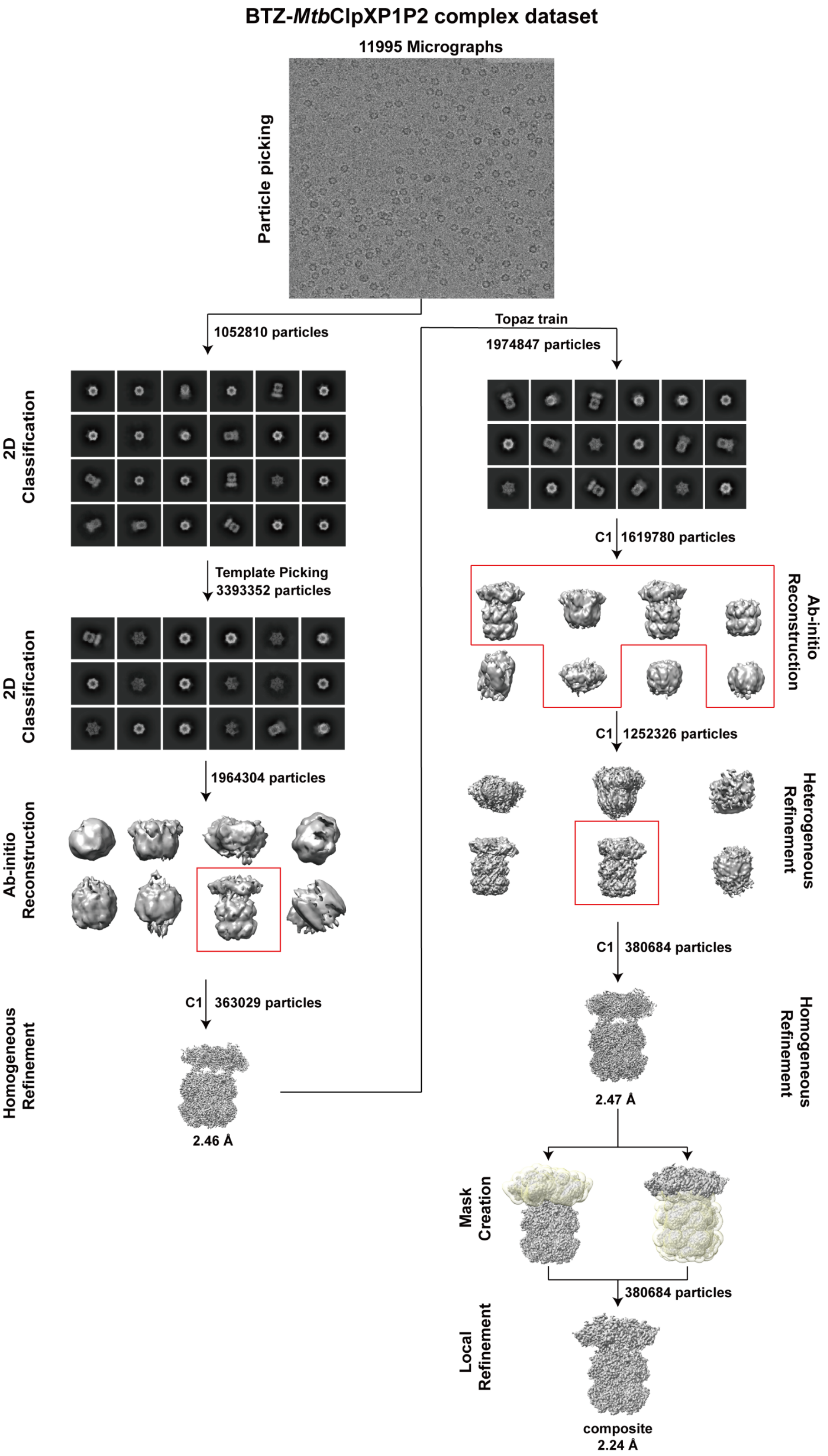

Supplementary Fig. 12 | Cryo-EM data processing pipeline for the BTZ-*Mtb*ClpXP1P2 complex dataset.

Data processing pipeline for the BTZ-*Mtb*ClpX1P2 complex dataset. The masks for subtraction are shown as transparent yellow surfaces. Red boxes indicate selected classes.

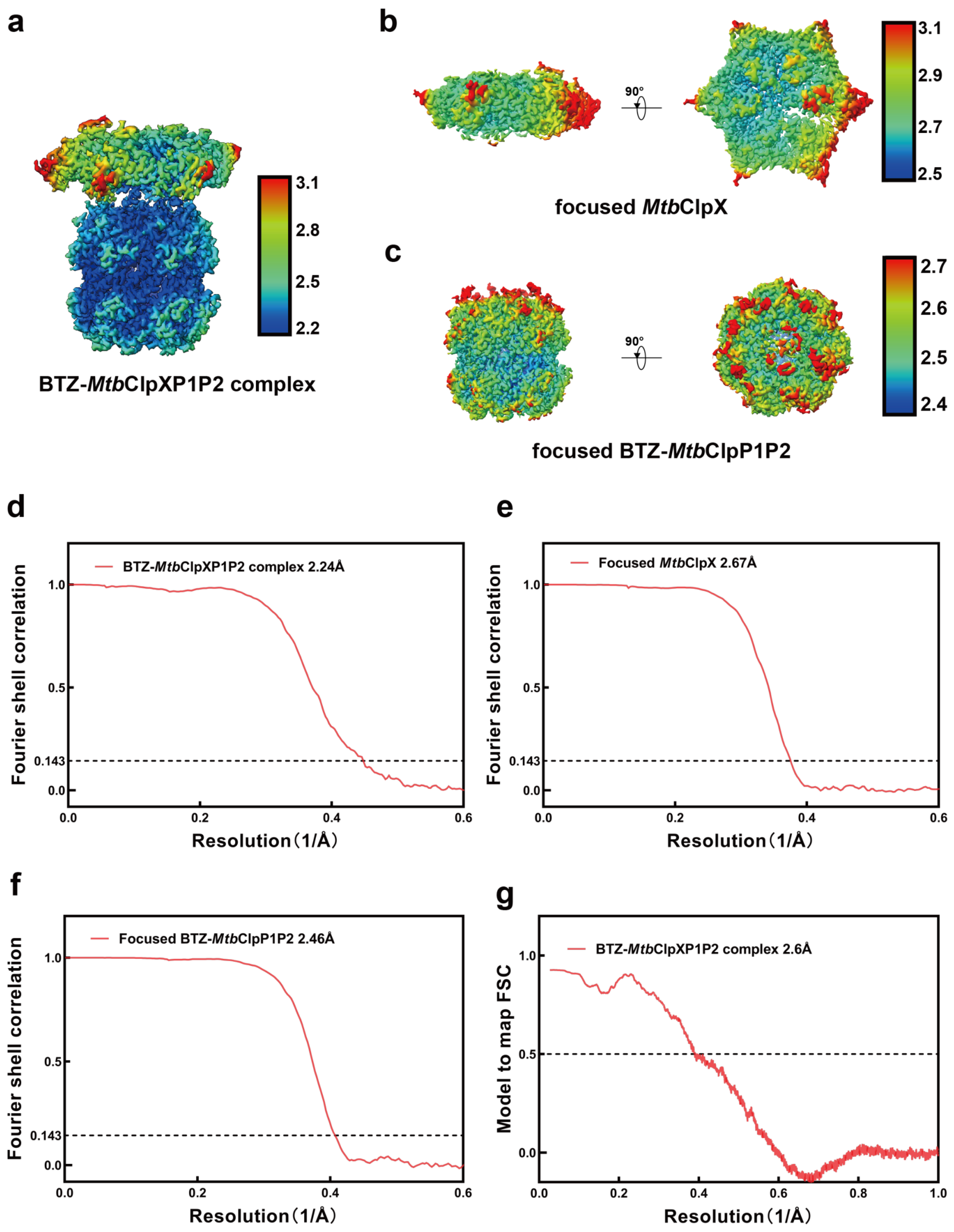

Supplementary Fig. 13 | Resolution assessment of the BTZ-*Mtb*ClpXP1P2 complex cryo-EM structures.

a, Local resolution assessment for the structures determined from the BTZ-*Mtb*ClpXP1P2 complex datasets. **b-c**, Local resolution assessment for the BTZ-*Mtb*ClpXP1P2 complex focused on *Mtb*ClpX **(b)** and BTZ-*Mtb*ClpP1P2 **(c)**. **d-f**, Global resolution assessment by Fourier shell correlation (FSC) at the 0.143

criterion. **g**, Correlations of model vs map by FSC at the 0.5 criterion.

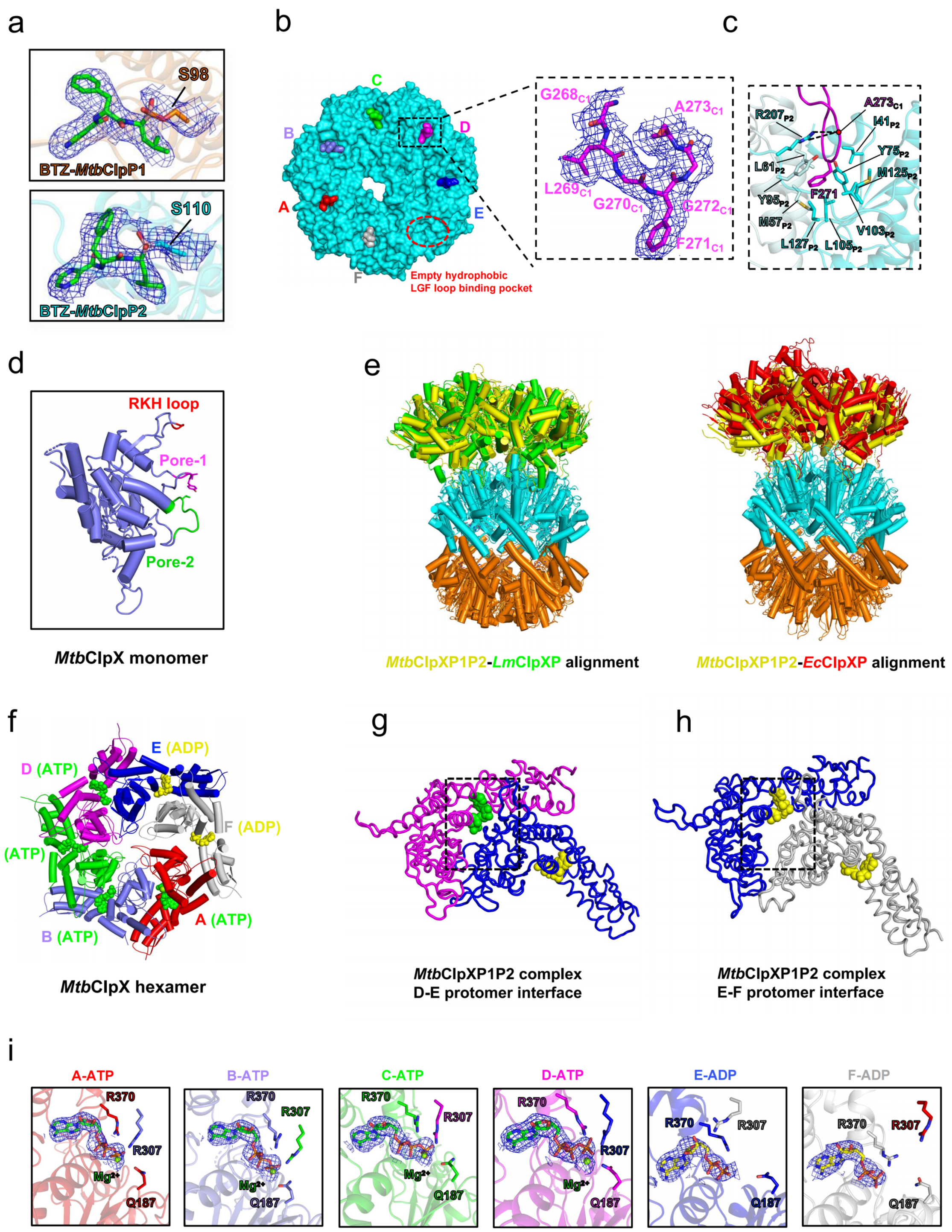

Supplementary Fig. 14 | Structural features of the *Mtb*ClpXP1P2 complex.

a, Blue meshes showing the densities of the active site bound BTZ in the *Mtb*ClpXP1P2 complex. **b**, Molecular surface showing LGF loops (colored by

protomer colors) bound to ClpP2 heptamer (left panel). Cryo-EM density of a *Mtb*ClpP2 bound *Mtb*ClpX LGF loop (residues 268–273) is shown in the black dotted box (right panel). **c**, A cartoon showing an LGF loop (magentas) bound within the *Mtb*ClpP2 LGF loop binding pocket. Residues involved in hydrophobic interaction or hydrogen bonding (dashed lines) are shown and labelled. **d**, Positions of the RKH loop (red), pore-1 loop (magentas), and pore-2 loop (green) in a ClpX monomer. **e**, Pairwise superpositions of the ClpXP complex in *Mycobacterium tuberculosis* (*Mtb*ClpXP1P2), *Listeria monocytogenes*[5] (*Lm*ClpXP) and *Escherichia coli*[6] (*Ec*ClpXP) using ClpP as the reference. **f**, A cartoon representation of the *Mtb*ClpX hexamer showing the nucleotide state of each ClpX protomer (ATP, green sphere; ADP, yellow sphere). **g-h**, Comparison of the *Mtb*ClpXP1P2 complex protomer interface between the D-E protomers in ATP bound **(g)** and E-F protomers in ADP bound **(h)**. **i**, Cryo-EM densities of the bound nucleotides in the *Mtb*ClpX hexamer.

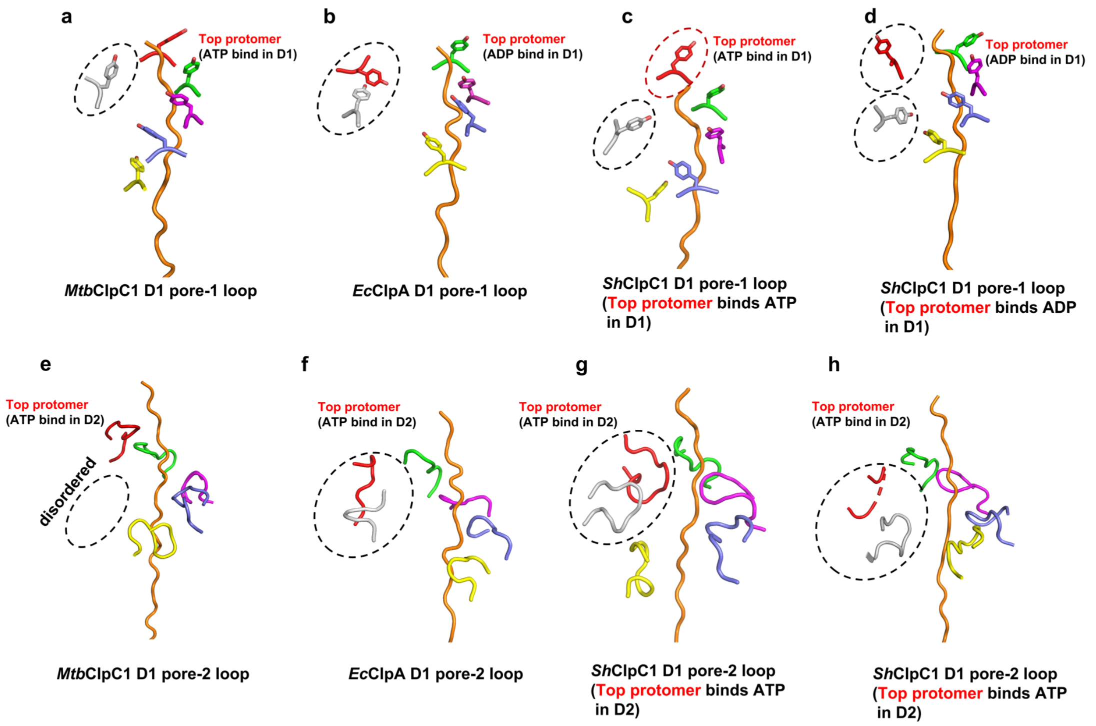

Supplementary Fig. 15 | Substrate interaction by *Mtb*ClpC1, *Ec*ClpA and *Sh*ClpC1.

a-d, Interactions between the substrate peptides (orange) and D1 pore-1 loops of *Mtb*ClpC1, *Ec*ClpA and *Sh*ClpC1. Conserved tyrosine residues in the D1 pore-1 loops of *Mtb*ClpC1 **(a)**, *Ec*ClpA (PDB code: 6W1Z)[7,8] **(b)** and *Sh*ClpC1 (PDB code: 8XOO and 8XON)[9] **(c-d)** in their respective holoenzymes are shown. In *Mtb*ClpC1, only the seam protomer D1 pore-1 loop does not interact with the substrate. In *Ec*ClpA and *Sh*ClpC1-conformation B (*Sh*ClpC1 top protomer D1 bound to ADP), *Ec*ClpA and *Sh*ClpC1 D1 pore-1 loops in both the top and seam protomers do not interact with the substrate. In *Sh*ClpC1-conformation A (*Sh*ClpC1 top protomer D1 bound to ATP), its seam protomer D1 pore-1 loop does not interact with the substrate, while its top protomer D1 pore-1 loop has a weaker interaction with the substrate, probably due to ATP binding. **e-h**, Interactions between the substrate peptides and D1 pore-2 loops of *Mtb*ClpC1, *Ec*ClpA and *Sh*ClpC1. In *Mtb*ClpC1, the D1 pore-2 loop of the top protomer interacts with the substrate peptide, whereas the D1 pore-2 loop of the seam protomer is not resolved, likely due to its flexibility. In *Ec*ClpA and

*Sh*ClpC1, the D1 pore-2 in both the top and seam protomers do not interact with the substrate.

Supplementary table

Cryo-EM data collection, refinement and validation statistics

	Apo MtbClpP1P2 (EMD-39164, PDB 8YD4)	BTZ-MtbClpP1P2 (EMD-61847, PDB 9JVZ)	BTZ-MtbClpP1P2 (EMD-39163, PDB 8YD2)	BTZ-MtbClpXP1P2 (EMD-39161, PDB 8YD0)	BTZ-MtbClpC1P1P2 conformation1 (EMD-39162, PDB 8YD1)	BTZ-MtbClpC1P1P2 conformation2 (EMD-39157, PDB 8YCX)	BTZ-MtbClpC1P1P2 conformation3 (EMD-61842, PDB 9JVP)
**Data collection and processing**							
Magnification	45000	165000	165000	165000	165000	165000	165000
Voltage (kV)	200	300	300	300	300	300	300
Electron exposure (e–/Å^2)	60	50	50	50	50	50	50
Defocus range (μm)	0.8-2.5	0.8-2.4	0.8-2.4	0.8-2.4	0.8-2.4	0.8-2.4	0.8-2.4
Pixel size (Å)	0.88	0.71	0.71	0.71	0.71	0.71	0.71
Symmetry imposed	*C7*	*C1*	*C7*	*C1*	*C1*	*C1*	*C1*
Initial particle images (no.)	1211953	2066604	2066604	1052810	1132003	1132003	1132003
Final particle images (no.)	256622	668234	668234	380684	95184	433061	520973
Map resolution (Å)	3.69	2.56	2.39	2.24	2.81	2.2	2.15
FSC threshold	0.143	0.143	0.143	0.143	0.143	0.143	0.143
Map resolution range (Å)	3.52-9.92	2.50-3.24	2.32-2.91	2.15-6.25	2.68-13.74	2.08-5.90	2.02-5.79
**Refinement**							
Initial model used (PDB code)	PDB 6VGQ	PDB 6VGQ	PDB 6VGQ	PDB 6VGQ/ de novo	PDB 6VGQ/ de novo	PDB 6VGQ/ de novo	PDB 6VGQ/ de novo
Model resolution (Å)	3.7	3.0	2.5	2.6	2.9	2.7	2.60
FSC threshold	0.5	0.5	0.5	0.5	0.5	0.5	0.5
Map sharpening *B* factor (Å^2)	-154.8	-78.4	-68.9	-28.1	-12.5	-13.3	-12.9
Model composition							
Non-hydrogen atoms	18753	19586	19586	36225	43497	46505	46966
Protein residues	2436	2506	2506	4631	5513	5892	5946
Ligands	0	14	14	22	31	32	34
B factors (Å^2)							
Protein	30.54	102.09	26.68	64.85	71.47	77.58	74.60
Ligand	--	33.40	21.61	49.48	36.69	39.68	26.90
R.m.s. deviations							
Bond lengths (Å)	0.001	0.004	0.007	0.012	0.011	0.011	0.012
Bond angles (°)	0.376	0.732	1.151	1.307	1.314	1.321	1.350
Validation							
MolProbity score	1.28	1.86	1.67	1.66	1.84	1.87	1.92
Clashscore	4.99	17.44	14.67	6.51	8.51	8.9	10.41
Poor rotamers (%)	0.71	0.60	0.35	0.21	0.95	0.77	0.99
Ramachandran plot							
Favored (%)	97.94	97.38	98.26	95.70	94.44	94.22	94.34
Allowed (%)	2.06	2.62	1.74	4.22	5.45	5.57	5.49
Disallowed (%)	0.00	0.00	0.00	0.09	0.11	0.21	0.17

Supplementary references

1 Zhang, T., Bishai, W. R., Grosset, J. H. & Nuermberger, E. L. Rapid assessment of antibacterial activity against Mycobacterium ulcerans by using recombinant luminescent strains. *Antimicrob Agents Chemother* **54**, 2806-2813 (2010). https://doi.org:10.1128/aac.00400-10

2 Gersch, M., List, A., Groll, M. & Sieber, S. A. Insights into structural network responsible for oligomerization and activity of bacterial virulence regulator caseinolytic protease P (ClpP) protein. *J Biol Chem* **287**, 9484-9494 (2012). https://doi.org:10.1074/jbc.M111.336222

3 Pahl, A. *et al.* Reversible Inhibitors Arrest ClpP in a Defined Conformational State that Can Be Revoked by ClpX Association. *Angew Chem Int Ed Engl* **54**, 15892-15896 (2015). https://doi.org:10.1002/anie.201507266

4 Kim, L. *et al.* Structural insights into ClpP protease side exit pore-opening by a pH drop coupled with substrate hydrolysis. *EMBO J* **41**, e109755 (2022). https://doi.org:10.15252/embj.2021109755

5 Gatsogiannis, C., Balogh, D., Merino, F., Sieber, S. A. & Raunser, S. Cryo-EM structure of the ClpXP protein degradation machinery. *Nat Struct Mol Biol* **26**, 946-954 (2019). https://doi.org:10.1038/s41594-019-0304-0

6 Ghanbarpour, A. *et al.* A closed translocation channel in the substrate-free AAA+ ClpXP protease diminishes rogue degradation. *Nat Commun* **14**, 7281 (2023). https://doi.org:10.1038/s41467-023-43145-x

7 Lopez, K. E. *et al.* Conformational plasticity of the ClpAP AAA+ protease couples protein unfolding and proteolysis. *Nat Struct Mol Biol* **27**, 406-416 (2020). https://doi.org:10.1038/s41594-020-0409-5

8 Kim, S., Fei, X., Sauer, R. T. & Baker, T. A. AAA+ protease-adaptor structures reveal altered conformations and ring specialization. *Nat Struct Mol Biol* **29**, 1068-1079 (2022). https://doi.org:10.1038/s41594-022-00850-3

9 Xu, X. *et al.* Structural insights into the Clp protein degradation machinery. *mBio* **15**, e0003124 (2024). https://doi.org:10.1128/mbio.00031-24
