# Supplementary material for: Structural Insights into Bortezomib-Induced Activation of the Caseinolytic Chaperone-Protease System in Mycobacterium tuberculosis
Source: Nat Commun. 2025 Apr 11;16:3466. doi: 10.1038/s41467-025-58410-4 (PMC11992174; doi:10.1038/s41467-025-58410-4)
Supplement: Supplementary file 2 — Reporting Summary [file 41467_2025_58410_MOESM2_ESM.pdf]

nature portfolio

Corresponding author(s): Xiaoli Xiong

Last updated by author(s): Mar 10, 2025

Reporting Summary

Nature Portfolio wishes to improve the reproducibility of the work that we publish. This form provides structure for consistency and transparency in reporting. For further information on Nature Portfolio policies, see our Editorial Policies and the Editorial Policy Checklist.

Statistics

For all statistical analyses, confirm that the following items are present in the figure legend, table legend, main text, or Methods section.

n/a	Confirmed	
☐	☒	The exact sample size (*n*) for each experimental group/condition, given as a discrete number and unit of measurement
☐	☒	A statement on whether measurements were taken from distinct samples or whether the same sample was measured repeatedly
☐	☒	The statistical test(s) used AND whether they are one- or two-sided<br>*Only common tests should be described solely by name; describe more complex techniques in the Methods section.*
☒	☐	A description of all covariates tested
☒	☐	A description of any assumptions or corrections, such as tests of normality and adjustment for multiple comparisons
☐	☒	A full description of the statistical parameters including central tendency (e.g. means) or other basic estimates (e.g. regression coefficient) AND variation (e.g. standard deviation) or associated estimates of uncertainty (e.g. confidence intervals)
☒	☐	For null hypothesis testing, the test statistic (e.g. *F*, *t*, *r*) with confidence intervals, effect sizes, degrees of freedom and *P* value noted<br>*Give P values as exact values whenever suitable.*
☒	☐	For Bayesian analysis, information on the choice of priors and Markov chain Monte Carlo settings
☒	☐	For hierarchical and complex designs, identification of the appropriate level for tests and full reporting of outcomes
☒	☐	Estimates of effect sizes (e.g. Cohen's *d*, Pearson's *r*), indicating how they were calculated

Our web collection on statistics for biologists contains articles on many of the points above.

Software and code

Policy information about availability of computer code

Data collection	EPU automated image acquisition software
Data analysis	CryoEM data processing (all available and referenced in methods): cryoSPARC v4.3.<br>Modelling/structure refinement/visualization (all available and referenced in methods): Coot v0.9.8.1; PHENIX v1.20.1; UCSF Chimera v1.16; UCSF ChimeraX v1.4.<br>Enzyme assay data analysis: Graphpad Prism v9.0.

For manuscripts utilizing custom algorithms or software that are central to the research but not yet described in published literature, software must be made available to editors and reviewers. We strongly encourage code deposition in a community repository (e.g. GitHub). See the Nature Portfolio guidelines for submitting code & software for further information.

Data

Policy information about availability of data

All manuscripts must include a data availability statement. This statement should provide the following information, where applicable:

- Accession codes, unique identifiers, or web links for publicly available datasets
- A description of any restrictions on data availability
- For clinical datasets or third party data, please ensure that the statement adheres to our policy

Cryo-EM maps of this study have been deposited in the Electron Microscope Data Bank (EMDB) with the accession codes EMD-39164 [https://www.ebi.ac.uk/

emdb/EMD-39164] (Apo-MtbClpP1P2), EMD-61847 [https://www.ebi.ac.uk/emdb/EMD-61847] (BTZ-MtbClpP1P2, C1 symmetry), EMD-39163 [https://www.ebi.ac.uk/emdb/EMD-39163] (BTZ-MtbClpP1P2, C7 symmetry), EMD-39161 [https://www.ebi.ac.uk/emdb/EMD-39161] (BTZ-MtbClpXP1P2), EMD-39162 [https://www.ebi.ac.uk/emdb/EMD-39162] (BTZ-MtbClpC1P1P2, conformation 1), EMD-39157 [https://www.ebi.ac.uk/emdb/EMD-39157] (BTZ-MtbClpC1P1P2, conformation 2) and EMD-61842 [https://www.ebi.ac.uk/emdb/EMD-61842] (BTZ-MtbClpC1P1P2, conformation 3). The atomic coordinates and structure factors for the structures determined in this study have been deposited in the Protein Data Bank under the accession codes 8YD4 [https://doi.org/10.2210/pdb8YD4/pdb] (Apo-MtbClpP1P2), 9JVZ [https://doi.org/10.2210/pdb9JVZ/pdb] (BTZ-MtbClpP1P2, C1 symmetry), 8YD2 [https://doi.org/10.2210/pdb8YD2/pdb] (BTZ-MtbClpP1P2 C7 symmetry), 8YD0 [https://doi.org/10.2210/pdb8YD0/pdb] (BTZ-MtbClpXP1P2), 8YD1 [https://doi.org/10.2210/pdb8YD1/pdb] (BTZ-MtbClpC1P1P2, conformation 1), 8YCX [https://doi.org/10.2210/pdb8YCX/pdb] (BTZ-MtbClpC1P1P2, conformation 2) and 9JVP [https://doi.org/10.2210/pdb9JVP/pdb] (BTZ-MtbClpC1P1P2, conformation 3). The PDB code of the previously published structure used in this study is 6VGQ [https://doi.org/10.2210/pdb6VGQ/pdb]. Source data are provided as a Source Data file.

Research involving human participants, their data, or biological material

Policy information about studies with human participants or human data. See also policy information about sex, gender (identity/presentation), and sexual orientation and race, ethnicity and racism.

Reporting on sex and gender	Not applicable
Reporting on race, ethnicity, or other socially relevant groupings	Not applicable
Population characteristics	Not applicable
Recruitment	Not applicable
Ethics oversight	Not applicable

Note that full information on the approval of the study protocol must also be provided in the manuscript.

Field-specific reporting

Please select the one below that is the best fit for your research. If you are not sure, read the appropriate sections before making your selection.

☒ Life sciences ☐ Behavioural & social sciences ☐ Ecological, evolutionary & environmental sciences

For a reference copy of the document with all sections, see nature.com/documents/nr-reporting-summary-flat.pdf

Life sciences study design

All studies must disclose on these points even when the disclosure is negative.

Sample size	No sample size calculation was performed as this study does not involve human or animal subjects.
Data exclusions	No data were excluded.
Replication	All experiments were performed with appropriate replicates, as described in the figure legends and Methods section.
Randomization	Not applicable or relevant, as no human or animal subjects were used in this study.
Blinding	Blinding was not necessary in this study since no subjective allocation was involved.

Reporting for specific materials, systems and methods

We require information from authors about some types of materials, experimental systems and methods used in many studies. Here, indicate whether each material, system or method listed is relevant to your study. If you are not sure if a list item applies to your research, read the appropriate section before selecting a response.

Materials & experimental systems

n/a	Involved in the study
☒	☐ Antibodies
☒	☐ Eukaryotic cell lines
☒	☐ Palaeontology and archaeology
☒	☐ Animals and other organisms
☒	☐ Clinical data
☒	☐ Dual use research of concern
☒	☐ Plants

Methods

n/a	Involved in the study
☒	☐ ChIP-seq
☒	☐ Flow cytometry
☒	☐ MRI-based neuroimaging

Plants

Seed stocks	N/A
Novel plant genotypes	N/A
Authentication	N/A
